# A quantitative validation of a qualitative phenomenological art-therapy cross-cultural study

**DOI:** 10.3389/fpsyg.2025.1530433

**Published:** 2025-04-24

**Authors:** Silvia Wyder

**Affiliations:** Rrosa Institute, Palermo, Italy

**Keywords:** art therapy research, qualitative methodology, phenomenology, quantitative research, house

## Abstract

**Introduction:**

This art therapy and cross-cultural research (France, Switzerland and Japan) investigated the topical suggestion of the ‘house’ in psychiatric settings via painting and drawing. The aim was to search whether this theme could serve as a symbolic representation of patients’ selfhood, and if it would allow pictorial, and, or verbal manifestation of Posttraumatic Stress Disorder phenomena. This paper focuses on presenting the developed mixed-methods study strategy and the obtained results.

**Methods:**

A qualitative phenomenological approach was applied in order to gain in-depth pictorial and narrative patient-based material through retroactive note taking from memory. Further data sets consisted of semi-structured interviews and closing discussions. A focus groups art therapy method was carried out focusing on individuals’ behavior, and group dynamics. A phenomenological coding procedure was applied following, allowing searching for emerging themes and patterns. A quantitative analysis was carried out by examining the phenomenological qualitative material, including semi-structured interview data. The study included 30 adult (out-patients) and adolescent (closed unit) patients (15-68 years).

**Results:**

Based on the carried out coding procedure seven overarching clusters could be identified such as ‘House/Home’, ‘Human Relationship’, ‘Inner Life’,‘Physical/Mental Health’, ‘Culture’, ‘Nature’, and ‘Aesthetic’. These clusters permitted highlighting similarities and distinctions between three socio-culturally and age-related diverse groups irrespective of the clinical setting. Matching cluster occurrence rates were observed in all three data sets in all three venues.

**Discussion:**

The findings attest a pronounced topical self-reference via ‘house’ paintings and drawings. Methodologically, the results demonstrate the validity of the carried out phenomenological qualitative research strategy in quantitative terms; this mixed-methods approach is thus an effective procedure in art therapy research. The result is particularly salient within the longest five months’ fieldwork study (Switzerland).

## 1 Introduction

In an era of globalization and multi-cultural societies, identifying the most suitable art therapies for mental health issues requires validated art therapy research approaches that need to be considered from broad methodological, and culture-sensitive perspectives. There is however a dearth of art therapy research, and, in particular, of research procedures that would be fitting for the multi-dimensional exploration that art therapy studies allow.

The present paper describes a specific but broadly applicable art-therapy research approach as well as the theory that substantiates it. The research reported on in this paper was part of an extensive approach to carry out a comparative art therapeutic study under controlled conditions in multiple clinical venues in Europe and Japan, and covers several research questions in parallel, among them the formulation of a coherent approach to research in art therapy, the suitability of a specific topic among different culturally and contextually diverse cohorts, the degree to which verbal and artistic expression signal a specific pathology (Posttraumatic Stress Disorder, PTSD phenomena), and several others. It was expected that a finer grained picture would require large amounts of diverse data, and that the chosen cohorts should exhibit a sufficient degree of variation that it would always be possible to construct a comparator from two venues when scrutinizing the third.

This article draws on a phenomenological approach to qualitatively investigate humans’ lived experience. By means of a novel approach, it introduces a subsequent quantitative overall analysis of the qualitative patient-specific findings to provide a cohort-level perspective. This dual approach allows to quantitatively discuss qualitative phenomenological findings, a procedure which is still relatively uncommon in art therapy research. It attempts to both propose a research methodology that is appropriate to art therapy research in multiple cultures and their various ethnicities, and to apply this approach to different clinical contexts. In doing so, a quantitative exploration of multi-facetted qualitative data was obtained through clinical art therapy fieldwork, which allowed illumination and exploration of a range of variables; art therapeutic, methodological, socio-cultural, topical, and age-related. Importantly, this procedure permitted a cross check of the different qualitative phenomenological and quantitative results stemming from the patients’ themselves, and the researcher.

In order for this to be feasible, a globally valid single topic has been sought to ensure comparability and validity of the individual clinical art therapy fieldwork settings. The chosen theme was that of the ‘house’, specifically, ‘the house as symbolic representation of the self’ (Wyder, 2023), which allowed topic-related cross-cultural investigations into psychiatric patients’ expressions of lived experience via their notions of habitat. Also, given the nature of a predominately phenomenological qualitative research approach it is worth noting that spatiality and dwelling, or in the present case the house, are important notions within phenomenology. The theme additionally served as a means of gathering fine-grained material for comparative purposes, be these related to, e.g., patients’ socio-cultural contexts, notions of self-representation, or possible depiction and narratives of Posttraumatic Stress Disorder (PTSD) phenomena.

Such an extensive cross-cultural art therapy study incorporating the theme of the house seems never to have been carried out. This concept has undoubtedly received more in-depth attention from philosophers (Bachelard, 1957, Heidegger, 1936-1953), psychiatrists (e.g. Klessmann and Eibach, 1993), psychoanalysts (e.g., Knellessen, 2012) and architecture theorists (e.g., Jormakka, 2003/2006/2007) than from art therapists, a motivation to investigate its potential to stand in for the self symbolically.

In the absence of similar topic-related investigations prior to this study, the study’s initial approach separated qualitative and quantitative elements. This comprised a qualitative phenomenological study, with a number of additional quantitative elements, covering several geographical and cultural contexts (Japan, Switzerland, and France). Fieldwork procedures consisted of clinical art therapy group workshops. More specifically, the obtained data sets included the qualitative observations based on patients’ narratives and aesthetic works (drawings and paintings), semi-structured interviews (Wyder, 2016), and discussions. Additional quantitative data regarding psychometric test results were also obtained but will not be discussed here, as they are not relevant to the topic of this paper. Only at a later point of the study did the need and opportunity for quantitative analysis of the qualitative data become compelling. Demonstration of the applicability and validity of the combined procedures forms the basis of the analysis of the obtained findings. Operational and methodological aspects will be covered in section 2.

The focus on phenomenology results from its suitability to art therapy practice and research: a phenomenological methodology allows the researchers and, or practitioners to experience and to observe the interaction with the patients. It additionally allows the integration of the concept of suspension, or epochē, which Spinelli (2005, p. 20) describes as [...] setting aside our initial biases and prejudices of things, to suspend our expectations and assumptions [...]’, notions which are of crucial importance in general, and even more so in cross-cultural, research.

While research into mixed-methods art therapy exists, a detailed analytical procedure that would lead from the phenomenological outcomes to quantitative results often remains at an abstract level, and concrete implications for phenomenology-based research are not commonly spelled out. More importantly, quantitative investigations of art therapy methodologies have focused on investigating its efficacy, but not on validating the phenomenological basis underlying this approach to art therapy. This paper discusses these aspects in depth, details the applied methodology, but also takes one step further by establishing a quantitative validation of the qualitative phenomenological approach as one of the specific outcomes of the research itself.

This paper thus focuses mainly on the applied research methodology and on its outcome. The objectives of this paper are multiple: firstly, given the paucity of prior mixed-methods art therapy research, this study was an attempt to propose, apply and evaluate a rigorous approach that would combine both phenomenological qualitative and quantitative methods. Secondly, given the multiple elements [e.g., fieldwork observational notes (patients’ narratives and aesthetic works), semi-structured interviews (Wyder, 2016), PTSD test procedures and so forth] of the applied mixed-methods approach, a cross-validation of different elements of the phenomenological qualitative and quantitative methods against each other is crucial. Specifically, phenomenological approaches have been difficult to validate; in the present case, the richness of expected data should shed light on the suitability and reliability of the applied method in general, and particularly in the context of art therapy research. Finally, by applying the same approach to multiple venues, a comparative investigation of the suitability of the proposed topic and approach should be evaluated.

Before coming to the details of the study, a brief discussion of the art therapeutic context and of the employed topic (the *house*), as well as the larger context of spatiality, is needed.

### 1.1 Art therapy

As there are several approaches to art therapy I will briefly outline my stance and definition, with its focus put on the symbolic material, which can emerge in patients’ aesthetic works and narratives.

In my view, art therapy can be considered as a form of psychotherapy that considers participants’ aesthetic expressions as equally important as the spoken word. Crucially, this approach involves the interaction of three essential elements – the patient, the aesthetic work, and the art therapist on equal footing. In other words, there exists according to Knill (2007, p. 62) ‘a form of ‘Dreierbeziehung’ (three-way relationship), with the aesthetic work literally becoming a player in its unpredictable and surprising process of creation’ (my translation from German). While art therapy needs to involve and reach patients’ self, direct access via speech is at times not only difficult but may even be counterproductive as it may trigger defense, or avoidance mechanisms. Instead, intercalating an intermediate layer (e.g., paintings), which can stand in symbolically for the self, may allow both art therapists and patients to address their lived experiences indirectly to conjointly express and achieve both ‘exterior’ and ‘interior’ perspectives.

In this research the third layer has been implemented in form of the proposed topic of the house in order to foster the emergence and the specific forms of aesthetic and verbal expressions regarding patients’ conscious and unconscious material. Crucially, art therapy offers the potential to explore such content ‘into sensuous forms’ (Jung, 1991, xvii) via individually created paintings, or other aesthetic forms (e.g., sculpture, dance) that are narrated by themselves, which are related to persons’ selfhood. In other words, the importance of the emergence of inner symbolic material through which personalized distinctions can become manifest within varying socio-cultural contexts is strongly emphasized, ideally leading to a process of individual change over time.

### 1.2 The topic of the house

The principal topic of this research was based on the *house* seen from the *outside.* Notions of what *house* and *home* consist of are diverse and depending on scholars’ definitions are regarded as being different or the very same. For example, *home,* following Heidegger (in Hasse, 2021, p. 426), ‘addresses living as a practice of life’ (my translation from German) and rather refers to a *process*, whereas *house*, through its architectonical physicality of built form (Buchli, 2013), refers to a *static object*. Crucially, the house theme allows addressing and expressing inner psychological states or experiences in reference to notions of the *outside and inside*, that is, material and, or emotive content.

Given the central role of the concept of the house in this study, some definitions are provided and the resulting findings will highlight the numerous psychological, societal, or conceptual aspects related to this notion even though the emphasis of this paper is on the methodological findings.

Relating to historic, but also to contemporary times, Buchli contextualizes the house, and more specifically architecture within ‘material culture’, in which the focus is put on the material qualities and the representative function of buildings. He defines the latter by highlighting its ramifications that rather refer to the house within its socio-cultural contexts, but which ultimately also point to its inhabitants. He writes (Buchli, 2013, p. 207):

‘This category of material culture was inextricably bound up in notions of social progress as with other technological forms but significantly: moral states of being. Portable artifacts could be divorced from their social and moral contexts, but not architecture. Built forms were almost always directly correlated with social and ethical forms. Cultures were compared to each other in terms of the social and technical complexity of their architectural forms, making architecture the single most significant artifact while describing the most fundamental unit of social organization: the family in its various guises’.

The family, the individual and their private house could thus be considered as an intimate microcosm, an original habitat, from which building-based material demonstrations thereafter emerged thanks to the symbolic usages they are able to attest (e.g., temples or state buildings). The present research thus relies on this physical symbolic space that can be created, depicted, filled, or narrated. Furthermore, bodily symbolic and architectural parallels could be envisaged as Henzler writes (Henzler, 2016, p. 265): ‘The house also has a symbolic reference to the body: the facade of the house can correspond to the outer appearance, the roof becomes the head, i.e., the consciousness (“upper room”), the basement the lower body, i.e., sexuality, drives and instincts, the unconscious assigned’ (my translation from German). Henzler thus symbolically refers to houses’ architectural spaces, or forms, that point, in her view, to specific body parts and their psychological attributions. Importantly, the overall focus and suggested topic of this research is based on the exterior physical, bodily form of the house, a containing shell, in contrast to notions of home with its emphasis on interiorities and domesticity.

### 1.3 Philosophical notions regarding house and space

Spatiality is a core concept of this research and of phenomenology. For this reason, some exploration of its groundings and its ramifications for art therapy are essential. Hence, I will address notions of space and by extension, the house, from a phenomenological perspective as, fundamentally, this concept serves, in my view, as an abstraction, as the overarching umbrella conception within which the notion of house is embedded.

The aim of this section is thus to establish a link between phenomenology, notions of space and the house, which serves as a means and resource through which persons’ life and world experiences became manifest during the art therapy clinical workshops. For instance, conscious and unconscious reasons for depicting an old traditional house in the country, in contrast to painting a house built of concrete in an urban situation, can be related to family histories, historical contexts, economic realities, personal choices regarding well-being, a higher significance given to traditional, socio-cultural and material grounded values, and aesthetic criteria, to name just a few. In that sense, depending on the reasons of choices made, these can point to persons’ idiosyncrasies within a specific socio-cultural environment that were expressed symbolically.

As pointed out earlier, notions of house and dwelling have been addressed by several philosophers with regards to the philosophical and symbolic potential, or the societal connotations these hold, and the personal forms that can be attributed with respect to houses’ interior, or exterior spaces, or a merger of both. For example, Heidegger (1936-53) addresses the relationship of human beings to spaces, living and building by highlighting that one cannot exist without these in an oppositional fashion, but rather that an inevitable intermeshing between these realms exists. Also Gaston Bachelard discusses symbolic components a house can incorporate with regards to reflections of the psyche. He wrote (Bachelard, 1957, p. 34): ‘The house is a body of images which gives man reasons or illusions of stability. We are constantly re-imagining its reality: to distinguish all these images would be to know the soul of the house; that would be to develop a veritable psychology of the house’. This quotation is especially useful to consider in the context of art therapy because of the symbolic function that the theme of the house incorporates, and the trigger function it can exercise in leading to patients’ idiosyncratic expressions. These can range from the cellar to the attic, from exteriorities to interiorities and structural characteristics such as statics and possible damage (e.g., as in trauma), or wholeness, thus offering patients a large range of verbal and pictorial expressions based on their lived experiences, or imagination.

## 2 Materials and methods

This section explores in greater depth the methodological framework, the research protocols and participant selection, as well as the analytical strategies employed.

### 2.1 Methodology and research method overview

#### 2.1.1 Methodology

The overall applied methodology draws on several approaches, or scholarship that may at times be similar, but also possibly divergent from each other. The reason for this is that this study integrated notions of phenomenology, that were most suitable for this piece of research, instead of following one specific methodology. More importantly, none of the existing methodologies allowed the particular mix of approaches that resulted ultimately in the validation outcomes this paper reports on.

#### 2.1.2 Method

Initially, as mentioned above, my research project was conceived around a mixed methods approach, building on a phenomenological qualitative stance, with an additional minor quantitative element to address psychometric testing (which will not be discussed in this paper). The integration of phenomenology here is important as its approach reaches far beyond what a given methodology implies; it is grounded within a specific strand of philosophy with its focus on the embodied experience of our existence. Given the complexity of human existence and the question of how to investigate it, but also, consequently, the amount of research material that art therapy allows to obtain (e.g., aesthetic works and narratives), phenomenologists such as, e.g., Merleau-Ponty, Gadamer or Fuchs (inter alia) provide the philosophical basis that this study built on.

As the subject matters in this study ultimately range from art therapy-related questions to philosophical notions, to socio-cultural comparative findings between European and Japanese participants, but also given the sizes of the individual data sets (with 30 patients overall), it became possible to extend a patient-by-patient qualitative approach with a higher level analysis and thus to additionally draw on a quantitative analysis of the phenomenologically analyzed fieldwork data.

Specifically, with tens of hours of interviews, hundreds of pages of field notes, and over 100 aesthetic works, a method to deal with such large data sets needed to be identified. The question thus arose how these phenomenological qualitative data could be analyzed and an overview achieved; a quantitative analytical procedure became compelling. As Klinke and Fernandez (2022) highlight, literature on methodological recommendations for such research is scant. They state: ‘The classical phenomenologists provide remarkably little in the way of concrete methodological advice. To develop an approach to collecting and analyzing behavioral evidence in phenomenological studies, we need to look to contemporary approaches to applied phenomenology and see which elements of these approaches might be adapted for our purposes.’ My procedure then was to integrate an interdisciplinary approach by drawing on diverse scholarship that seemed suitable. In particular, coding (van Manen, 2011) procedures (section 2.3) appeared particularly appropriate to extract - again using a phenomenological approach to the obtained qualitative data - fine-grained observations but in a way that lends itself to clustering, and thus in fine to quantification.

#### 2.1.3 Ethics statement

Ethical approval has been obtained by the University of Derby’s ethics’ committee in accordance with their guidelines before the start of the three clinical fieldworks. Subsequently, for each of the research locations in Europe and Japan a separate approval was sought. A further step consisted of the verification by the patients’ treating psychiatrists prior to submitting the consent form to the former. Patients’ art therapy workshop participation was voluntary. Patients’ names have been anonymized to safeguard their privacy. Depending on the languages spoken at the fieldwork locations, translations into German and French were carried out by the researcher. The translation from English into Japanese was carried out by a Japanese scholar of the University of Geneva in Switzerland. Written informed consent was received from all the patients including permission to publish photographs of their drawings and paintings. The aesthetic works remained the property of the patients. There were no animals involved in this study.

#### 2.1.4 Self-reflexivity

Embracing a self-reflexive stance is of crucial importance whilst carrying out qualitative phenomenological and quantitative research. For instance, for Kelly et al. (2017, p. 1) ‘[r]eflexivity has been defined as “critical self-reflection on how the researcher’s background, assumptions, positioning, and behavior impact on the research process” (Finlay and Gough, 2008, p.ix).’ In my view, the absence of self-reflexivity or being unconsciously led by one’s own subjectivity can result in procedures that are influenced by researchers’ own scientific expectations, or socio-cultural background, potentially impacting all the steps involved, starting from the formulation of a specific research question to the choice of the research methodology.

For example, there were several functions I held simultaneously during the fieldwork-related research, that of patients’ art therapist, the art therapy researcher, my own self-reflexive approach, all of which might have had a mutual influence. Furthermore, depending on the cultural context my ethnicity, and the fact that I am not part of the clinics’ staff, might have played a role regarding the ways, or the degree to which patients have opened up to me, and how I might have reacted towards them. Such methodological issues need to be taken into account as best as possible so as to become self-aware in order to establish findings that are as uninfluenced and as objective as possible. Finally, the researcher underwent regular supervision.

### 2.2 Participants and context

The participants to the study that took place from 2016 to 2018 in three psychiatric clinics in Paris, France (Hôpital Pitié-Salpétrière), Psychiatrie St. Gallen, Wil, Switzerland, and Ohmiya Kousei Hospital, Japan, constitute three separate cohorts. There were six participants at the Salpétrière, seven participants in Wil and 15 participants at Ohmiya. The duration of the art therapy fieldwork varied depending on the psychiatric clinic: in Wil the maximum patients’ attendance was of 5 months, in Paris it was 3 months, and in Omiya it was of 4 weeks (Table 1).

Overall, the three patient groups with an almost equal number of female and male patients consisted of a total of 30 patients. The age group spanned from adolescents to adults, that is, from 15 to 68 years. The adult cohort (Wil and Ohmiya) consisted of out-patients, whereas the adolescents resided in a closed ward (Paris). Psychopathology ranged from, for example, depression, Posttraumatic Stress Disorder, self-harm, psychosis, anxiety disorders, to substance use disorder.

Inclusion criteria were based on psychiatrists’ judgment regarding patients’ psychopathology and current mental situation, and their opinion on who the considered fit enough to partake in the study and to be confronted with an unknown person, a researcher. Exclusion criteria were thus that patients who were considered too fragile would not be able to be part of the art therapy study. Importantly, within this group of ‘selected’ patients at the three clinics, it was the patients themselves who ultimately decided if they would wish to participate in the study, to stay on, or to leave.

### 2.3 Art therapy method and data collection procedures

The carried-out protocol was the same in every fieldwork albeit adapted to the site-specific spatial and cultural conditions. Each venue’s workshop initiated with a short introduction of the theme of the house by displaying 31 postcards (of paintings, photographs, drawings, prints and a video-still) showing houses in various countries and cultures, which could serve (or not) as a visual, or thematic springboard for the participants.

In all research locations, the modalities were painting and drawing. The art material consisted of acrylic colors, gouache, pencils, colors, graphite, colored markers, rulers, erasers, graphite sticks, brushes, Japanese ink, ink stones, and paper sizes of A3 and B2 (and a paper roll, Wil). These were spread out in the same way in each psychiatric clinic during every art therapy session for patients’ unhindered choice. This retention of the art materials’ display including tables, chairs, or benches, may have provided patients with a sense of continuity and of a safe environment while additionally maintaining methodological rigor. No art teaching was involved.

Patients freely chose their seats at tables (or to stand in Wil) whilst painting, or drawing. During each session I verbally interacted with each patient individually in all the clinics by addressing their comments, and by asking questions related to their paintings and drawings. Overall group discussions occurred as well, to which patients could take part if they wished. Languages used were Swiss German, French, Japanese and English; no interpreter was present during the sessions.

Sessions’ closures usually involved verbal exchanges and cleaning of the art materials and tables. The aesthetic works were safely stored by the researcher and remained patients’ property after the end of the fieldworks. After each workshop patients’ works were photographed for documentation, thereby witnessing individuals’ process-related evolution, after ethical approval and informed written consent was received. Observational field notes based on patients’ verbal expressions about their aesthetic works and narratives, including their own narratives or paintings’ interpretations, personal life experiences, group interaction, bodily expressions and so forth, were written down from memory after each session outside the clinics.

Lastly, I conducted a 29-item individual semi-structured interview (Wyder, 2016) (recorded) with most participants in presence of their drawings and paintings at the end of each clinical fieldwork. Questions were geared to better understand patients’ experiences regarding, e.g., their perception of space and self, their creative process, and cultural notions in connection to the theme of the ‘house’. Some patients did not wish to take part in the interview, were ill, or no longer attended the clinic; the data is thus incomplete. During the semi-structured interview in Ohmiya, based on the clinic’s requirements, the interview length had on the one side to be shortened to a maximum of 30 min, and thus only 12 questions could be asked. The clinic’s requirement was that a nurse in one group, and a psychologist in the other were present during the interview (both of whom were familiar, and interacted, with the patients), which might have influenced patients’ answers. Finally, in Wil, an unstructured discussion was carried out with each patient in presence of their art works after the end of the study, which was not the case in the other two clinics.

Methodologically, all the above-described measures were maintained throughout each clinical fieldwork in order to sustain a systematic and scientific as possible procedure.

Specifically, the data collection process reported here begins with phenomenological observational written material of all patients’ during their clinical art-therapeutic activities. It also includes patients’ narratives, and their meaning-making of their aesthetic works in presence of myself and among the other patients. Semi-structured interviews (Wyder, 2016) were carried out individually with each patient. Also, a final discussion in presence of their aesthetic works was led in the case of Wil. In a second step, these data were phenomenologically scrutinized (coded) by adopting van Manen (2011) procedure, resulting in thematic material that was, in a third step, quantitatively assessed, namely through the rates of occurrences of the emerged themes, leading to patterns and, finally, to overarching clusters.

Concretely, the following multi-level phenomenological qualitative and quantitative data collection procedures have been carried out during the fieldwork at the three locations:

1. Phenomenological observations of patients’ lived experience within three clinical settings via their painting and, or drawing activities, including their narratives linked to the topic of the ‘house’. Observation of each patient’s interaction with the researcher, including group dynamics. Note taking from memory after each session of each patient (aesthetic works, narratives, interpersonal interaction, and bodily expressions) outside the clinics. Photographic documentation of drawings and paintings after each session (after received ethical informed consent). Researcher’s integration of a self-reflexive stance.
2. Semi-structured interview in German, French, English and Japanese (Wyder, 2016, recorded, transcribed and translated (if applicable)).
3. Psychometric PTSD and mental health test procedures (not discussed in this paper).
4. Final individual discussion (recorded) in presence of patients’ aesthetic works (Wil only), transcription.

#### 2.3.1 Semi-structured interview

The semi-structured interview (Wyder, 2016) including 29 questions, has been approved by the University of Derby’s ethics committee. It was administered individually and was recorded with the participants’ agreement. The main topical areas served to address the following notions:

- Trauma/PTSD
- Mental States
- Creative Process
- Materiality
- Perception of Space
- Culture

The semi-structured interview approach was chosen since, in-line with Bryman’s view (Bryman, 2012, p. 13), ‘the interviewee has a great deal of leeway in how to reply [...] given that the interview process is flexible’. The aim of this interview was to obtain supplemental or intersecting material, which could be complementary to the patients’ aesthetic works and narratives, and, or my own observations, and the psychiatric diagnosis, obtained retroactively as per my request.

#### 2.3.2 Analysis methods

**Figure 1:**
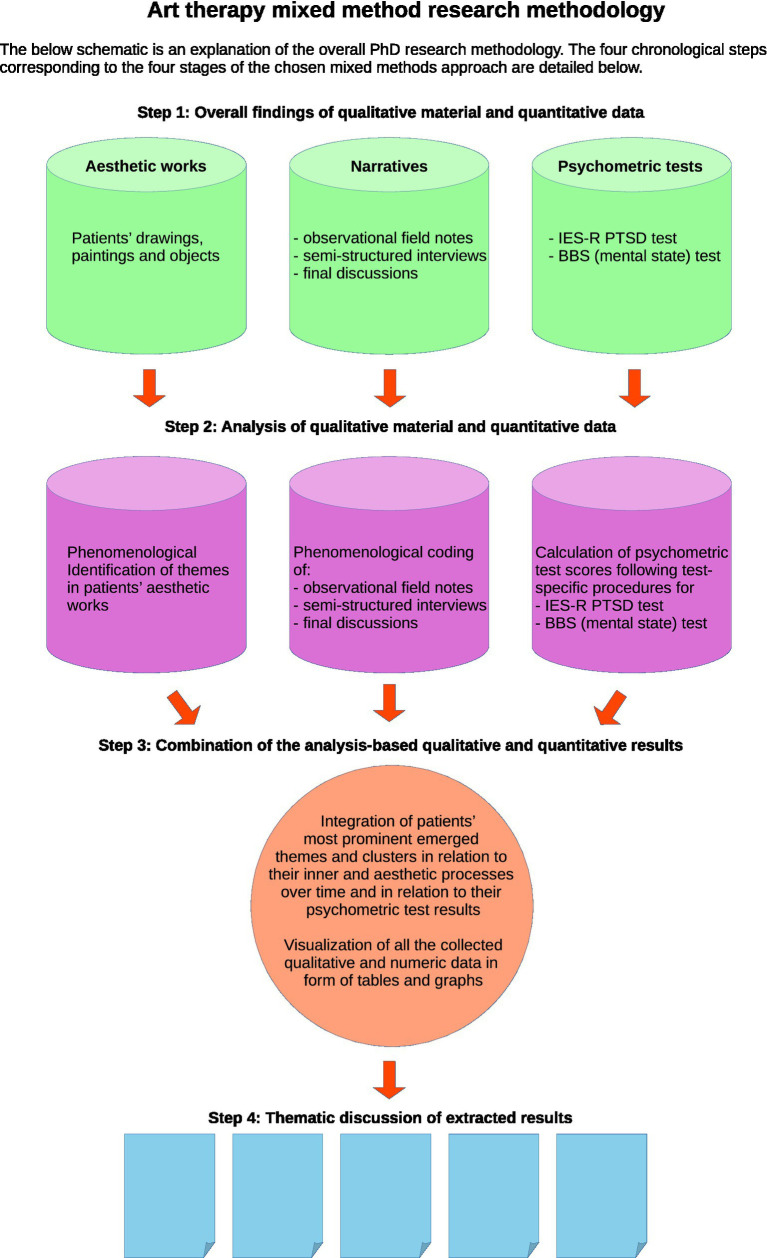
Schematic representation of the overall research strategy, Wyder, 2023.

**Figure 2:**
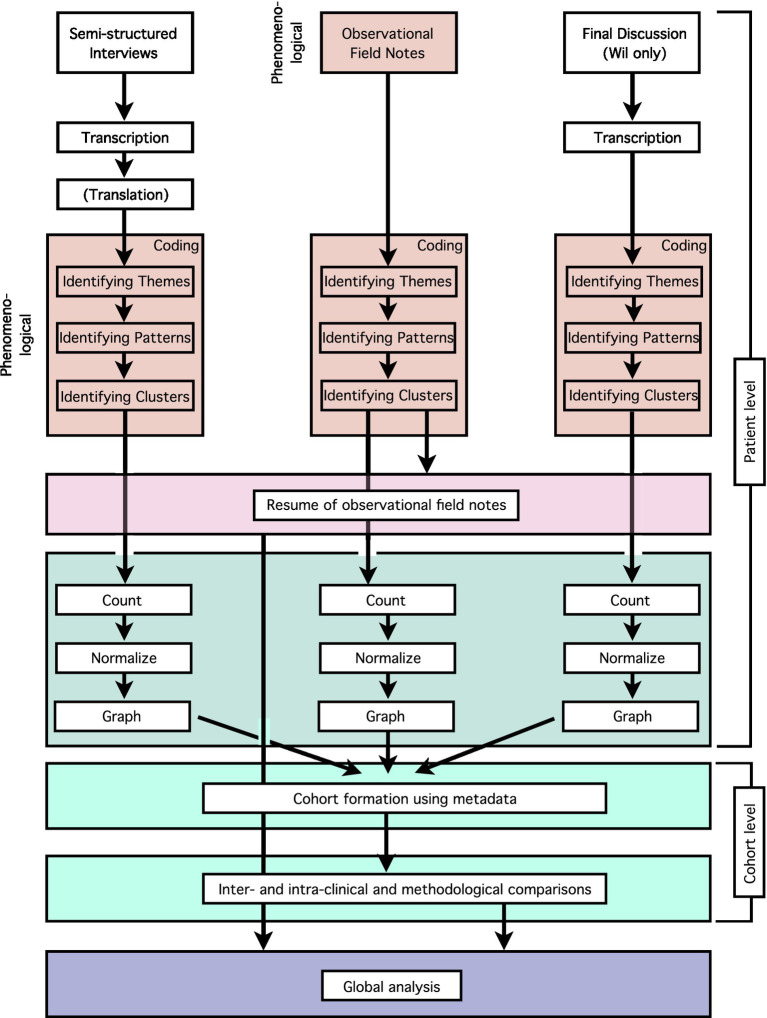
Analysis flow chart indicating the different steps (dark pink: phenomenological; light pink: qualitative; light blue: quantitative; dark blue: combined) of the analysis of the data for each patient. Pooling of the patient level data by incorporation of the patient meta-data allowed cohort-level (e.g., inter- and intra-clinical or methodological) comparisons.

The following summary consists of a compact overview of the data sets, and of the description of the analysis method (Figures 1, 2). The detailed numbers of the different types of data sets are presented in Table 1, section 4.2.

In brief, for each patient, the raw data consisted of:

1. Data set type 1 = Semi-structured interviews in English, French, German and Japanese (Wyder, 2016, recorded)
2. Data set type 2 = Observational field notes (written retroactively outside clinics)
3. Data set type 3 = Final discussion (recorded, Wil only)
4. Data set type 4 = Metadata (age, gender, location, type of clinic, clinical diagnosis)

Recorded audio data (data type 1 and 3) were transcribed and translated where needed. The description of the analysis procedures applied to these *patient-level* data forms the focus of this section, while the analysis at the cohort level will be addressed in the following *Result*s section.

To process these disparate data, and provide patient-specific quantitative descriptions, the following procedures were applied to the above data sets 1–3:

1. Application of van Manen (2011) coding procedure in order to phenomenologically search for emerging themes and patterns based on the researcher’s phenomenological penned down observations, and the recorded semi-structured interviews (Wyder, 2016) and discussions, for each patient individually, from which overall individual (quantitative) thematic clusters emerged.
2. Combination of these patient-specific (quantitative) patterns into larger cohort-level and cohort-specific quantitative characteristics.
3. Contextualization within these cohort-specific frameworks of the individual patient’s post-session phenomenological observational notes, including pictorial, and, or psychological shifts (if applicable) over time becoming manifest in their paintings, and, or narratives.
4. Adding up of the individual patient-specific themes and patterns into cohort-level patterns.
5. from these, establishing cohort specificities, with the resulting possibility of comparison between cohorts.

Concretely, the overall analytical strategy included qualitative phenomenological and quantitative procedures. The carried out qualitative procedures consisted of scrutinizing and phenomenological coding the phenomenological observational field notes (written down retrospectively from memory, including patients’ narratives and self-reflexive observation), material from patients’ recorded, transcribed and translated semi-structured interviews (Wyder, 2016), and patients’ aesthetic works (paintings and drawings) for each patient separately. Further, interactive group dynamics, participants’ mental and, or physical states, use of space and selected art, and other materials, as well as integrating possibly occurring socio-cultural specificities were taken into account.

The following graph shows a Schematic Representation of this Overall Research Strategy (Figure 1) leading to the Final Analysis [red round form at the bottom of the flowchart (Wyder, 2023)]. This served as a visual basis for the findings, which allowed elaboration and discussion in general as well as individuals’ similarities and divergences concerning house-related aesthetic and verbal content, patients’ mental health issues, and socio-cultural distinctions.

#### 2.3.3 Quantitative approaches to phenomenological data

As applying a quantitative approach to phenomenological data is non-standard, particularly in the field of art therapy, some contextualization is required. Mixing a phenomenological qualitative approach with a quantitative statistical analysis can be challenging, as art therapy qualitative phenomenological research that additionally integrates quantitative approaches is still uncommon. If quantitative measures are applied they mostly consist of psychometric tests, as for example Levenberg (2019) thesis with a focus on the use of quantitative measures [e.g., Beck’s Depression Inventory (BDI-II)] in art therapy demonstrates. In this sense, the methodology of the present art therapy research is rather uncommon as it integrates qualitative phenomenological methods together with quantitative procedures and presents phenomenological qualitative-based findings as quantitative results. To my knowledge, the most prominent use of quantitative statistics within art therapy research was carried out by Rose (Ruth) Ehemann, whose ‘Virtual Reality Therapie’ (Ehemann, 2012) study did not, however, embrace a phenomenological stance.

Furthermore, integrating phenomenology into qualitative research in, for example, psychology or therapy-related fields, can also be demanding for methodological reasons as Giorgi and Giorgi (2003, p. 244) point out: ‘Most of the major philosophers in the continental tradition strongly believed that phenomenological philosophy could help psychology in diverse ways. [...] Husserl believed that this approach could help clarify the fundamental concepts of psychology, and as a consequence, psychologists would be able to use the concepts consistently and more accurately. [...] However, these analyses were conceptual and philosophical even though they are very helpful for those psychologists who would adopt a phenomenological perspective. How one would apply the phenomenological method in psychology is not detailed.’ Even though the present study is situated in the realm of art therapy and not psychology, philosophical and methodological discussions may arise as the former is additionally related to the arts and is thus neither psychology, nor phenomenology proper. Specifically, art therapy per se, but also research in that field draw on several philosophical traditions and approaches but do not, in my view, necessarily share a common framework. For example, Andrea Gilroy (2006, p. 81) claims regarding art therapy research that it ‘enable[s] a deeper understanding’ than ‘quantitative methods [that] consider the *amount* of things’ that integrate a ‘positivist approach’. In contrast, she highlights that ‘qualitative research draws on phenomenological and ethnographic research traditions that enable a reflexive, heuristic approach in naturalistic settings’. Thus, even though the history and origins of art therapy are rather interdisciplinary in itself as it draws on scholarship of, e.g., the arts, psychological psychotherapy (namely C.G. Jung), psychoanalysis, phenomenology, socio-cultural and feminist approaches to name some, there still exists a tension regarding what kind of research methodology to be applied, and even more so in regards to integrating quantitative procedures.

To come back to this study’s specifics: the diverse clinical, socio-cultural, patients’ age-based, and geographic situations underscore the importance of implementing dedicated methodological procedures. As highlighted before, the rationale for the carried out research method mostly resides in the inductive search for possible emerging themes that would lead to patterns by investigating independent variables within the patients’ aesthetic works, their narratives and in relation to their socio-cultural environment that lead to a novel conception. In that sense, the initially foreseen analytical strategies were expanded in order to better understand and address the specific and varied data sets and material that emerged during the art therapy research process. My procedure is in partial agreement with Gerber et al.’s statement regarding arts-based research even though they rather refer to qualitative methods: ‘ABR [arts-based research] while embodying pluralistic ontologies and eclectic epistemologies, simultaneously resists rigid classifications and methodologies. ABR exists on a dynamic “dialectical edge” (Israelstam, 2007, p. 591) juxtaposing creativity and destruction, reason and imagination, conscious and unconscious, known and unknown (Chilton et al., 2015; Gadamer, 2007 (1992)).’ [...] ‘Arts-based research transcends disciplinary silos, cultural divisions, and other man-made societal and geo-political boundaries engaging multiple audiences in meaningful, transformative social discourse’ (Gerber et al., 2020, pp. 2, 5, 9). In line with Gerber et al.’s call for an interdisciplinary approach to research, which can be extended to integrate also quantitative approaches, a dichotomous research approach of ‘either’ (qualitative, and, or phenomenological), ‘or’ (quantitative) seemed inappropriate for this study, particularly given contemporary societies’ multi-cultural and ethnic complexity in general, with their high degree of heterogeneity that is reflected in clinical situations. Additionally, there remains the question in principle of *how to* come to terms with research findings that would rely on an exclusively descriptive account of complex studies, or with the question of whether an analytical statistical strategy via graphical presentations of the results would not benefit the general art therapy research community?

#### 2.3.4 The qualitative phenomenological approach

Since phenomenology is a philosophical endeavor, applying it to research requires some explanation in terms of the integrated philosophy itself, as it informs on the background of this study. For example, this research’s overarching philosophy is grounded in a phenomenological qualitative approach and follows Carel (2016, pp. 1, 2) who states: ‘Studying lived experience is not a variant form of scientific enquiry, but a method for examining pre-reflective, subjective persons’ experience as it is lived prior to its theorization by science’. In other words, the fundamental basis for human based research in the field of phenomenology-related art therapy situates itself in the in-depth observation of, and interaction with the patients through their embodied experiences and aesthetic manifestations.

Fundamentally, the phenomenological procedural method is based on Husserl’s phenomenological stance regarding human-based research as is explicated in the subsequent paragraph (Encyclopaedia Britannica, 2021):

‘The term was employed in the 20th century by Edmund Husserl, the founder of phenomenology, who saw it as a technique, more fundamental than that of abstraction and the examination of essences, that serves to highlight consciousness itself. The philosopher should practice a sort of Cartesian doubt, methodic and tentative, in regard to all commonsensical beliefs; he [she] should put them, and indeed all things of the natural-empirical world, in “brackets,” subjecting them to a transcendental suspension of conviction — to epochē. Without ceasing to believe in them, he [she] should put his belief out of action in order to focus upon the sheer appearances of houses, trees, and people, which then become tantamount to the existence of his awareness of them.’

Further, the phenomenological approach applied herein is linked to Spinelli’s (2005, p. 19) reference to Husserl’s original procedure who ‘argued it was to be the principal task of phenomenology to find the means to strip away, as far as possible, the plethora of interpretational layers added to the unknown stimuli to our experience in order to arrive at a more adequate, if still approximate and incomplete, knowledge of ‘things themselves.” Therefore and by following Spinelli (2005, p. 20) the methodological [...] epochē urges us to impose an ‘openness’ on our immediate experience [...] so that our subsequent interpretations of it may prove to be more adequate.’ Practically, my stance and method of patient-related interaction, observations as well as the subsequent note taking from memory were grounded in this phenomenological approach in which the notion of ‘openness’ (Spinelli, 2005) was sought.

In addition, Merleau-Ponty’s phenomenology is particularly pertinent as he considers human beings’ embodied experiences that is of corporeality, as primordial, an approach that is in opposition to the, e.g., Cartesian body–mind split, an exclusive emphasis on the intellect, or ratio. Hence, Merleau-Ponty’s approach integrates internal and external realities on equal footing, these constituting not a separate, but rather one single integrated structure of being. For example, Toadvine (2019, URL) referring to Merleau-Ponty writes: ‘[...] Merleau-Ponty radically distinguishes his project from that of both Descartes and Kant whom he claims have detached the conscious subject from the world that is given in experience.’ [...] ‘Merleau-Ponty insists that when speaking of the physical, the vital and the human structures, one should not conceive of them as acting on one another in a causal manner’. Therefore, the emphasis of this research was put on individuals’ bodily lived experiences and how and why these were expressed, and became manifest over time within their respective socio-cultural contexts.

More precisely, Angehrn expounds on Merleau-Ponty’s phenomenology regarding the developed concept of corporeality and methodological reduction as follows (2014, p. 210):

‘The theme of corporeality is considered a hallmark of Merleau-Ponty’s phenomenology, which, however, defines less an additional theme than a changed perspective and a specific deepening of the phenomenological reduction. Merleau-Ponty’s phenomenology exemplifies a theory of incarnated meaning, whereby incarnation affects both the side of the meaning structure as well as that of the subject and the shaper of the constitution, reception and communication of meaning. The embodied meaning as the mediation of soul and body, idea and materiality corresponds to the methodological approach of phenomenology, which Merleau-Ponty defines in the early main writings on the twofold defense of empiricism and intellectualism, in its place the persistent mediation from the interior and exterior. In order to grasp phenomena in their meaningfulness, it is not simply a matter of replacing the materialistic-causal description from the outside with the internal first-person perspective. Rather, it is important to open up the interweaving of inside and outside in which the real meaningfulness of experience emerges and in which the person already participates thanks to their mental and physical condition.’

It is precisely Merleau-Ponty’s (1945) notion of methodological oppositions regarding ‘empiricism’ and ‘intellectualism’, that is, of the ‘interweaving of inside and outside’, as Angehrn (2014, p. 210) puts it, that allows, on the one hand establishing a parallel to this research’s applied methodology by integrating qualitative phenomenological experientially based procedures. On the other hand, it also symbolically permits to establish a link to the theme of the house, as it literally incorporates notions of inside and outside, and which makes this topic such an all-encompassing and powerful tool to investigate persons’ idiosyncratic experiences.

#### 2.3.5 The quantitative analysis of phenomenological data through coding

A quantitative analysis of the qualitative data was not initially foreseen, but emerged naturally from a phenomenological analysis of the three qualitative data sets obtained for each of the three clinics. Indeed, the three cohorts were large enough (see Table 1) that it became possible to extract cohort-level information, allowing, e.g., on the one hand a cross-cultural comparison between the European and Japanese patients’ specificities, and on the other to highlight individual’s, and in-group distinctions.

The phenomenological coding procedure has been applied to my observational field notes with the aim to extract firstly patients’ individual themes, to be followed by grouping those into the overall three clinical, and socio-culturally diverse patient groups’ findings, based on a coding approach developed by van Manen (2011). This procedure led to the emergence of patterns that were grouped into overarching clusters. Even though these results are statistically valid, they must be regarded as indications pointing to patient-related phenomena and denotation that occurred during the research process, and not as ‘hard’ facts, or ultimate ‘truth’. van Manen (2011) explicates this coding process as follows:

‘[...] “analyzing” thematic meanings of a phenomenon (a lived experience) is a complex and creative process of insightful invention, discovery and disclosure. Grasping and formulating a thematic understanding is not a rule-bound process but a free act of “seeing” meaning. In other words, phenomenological themes are not objects or generalizations; metaphorically speaking, they are more like knots in the webs of our experiences, around which certain lived experiences are spun and thus lived through as meaningful wholes.’ [...] ‘However, the thematic meanings of human experience are self-constituted. They reflect the ways that we tend to make sense of life as human beings – as human beings who are embedded within certain linguistic, historical and cultural contexts. That is why we can say that human meanings are discovered but also self-disclosing, constructed by us but also constructed of us.’

Regarding van Manen (2011) statement that ‘phenomenological themes are not objects or generalizations’ I partly depart from his approach. In my view, I would argue that since he *does* speak of ‘knots in the webs of *our* (my italics) experiences’, and by reference to the notion of socio-cultural embedment, a certain level of generalizations is not only possible, but also, in the case of research, clinically useful as it allowed a more global understanding of the findings of this study (see below, section 3).

In particular, Sloan and Bowe explicate van Manen’s approach and the potential it bears thanks to hermeneutics. They refer to Hans-Georg Gadamer’s notion of interpretation and understanding of symbolic meaning, which is of special importance in an art therapy context as it includes narratives and aesthetic works. They write (Sloan and Bowe, 2014, p. 1295):

‘More recently, Max van Manen has been developing the hermeneutic approach of phenomenology. His approach follows Gadamer as his philosophy is that language reveals being within some historical and cultural contexts, understood by participants and researchers and through language, such as the language of the interview (Langdridge, 2007). Max van Manen’s hermeneutic phenomenology can be used to clarify phenomena in the fields of, for example, pedagogy, psychology and nursing in a practical way. He has stated that phenomenology formatively informs, reforms, transforms, performs, and pre-forms the relation between being and practice (Manen, 2007). This suggests that hermeneutic phenomenology has been evolving from a philosophy to a methodology.’

Whether Gadamer would agree that his approach is considered to be a ‘methodology’, or a method, remains open. Crucially however, the importance given to art, and by extension to art therapy in Gadamer’s philosophy is that it is not limited to language, the exclusivity of humans’ verbal expressions, but most importantly that he regarded works of art as equally relevant as a form of human expression, and similarly valid in order to gain a different kind of knowledge (see, e.g., ‘Die Aktualität des Schönen’, Gadamer, 1974).

#### 2.3.6 Analytical data procedure

The analysis itself was implemented to proceed in two steps:

A first step consisted of phenomenological coding of the data sets (1) to (3) resulting in qualitative phenomenological identification of themes and quantitative occurrences of the specific themes (‘tags’, see section 3.1).

**Figure 3:**
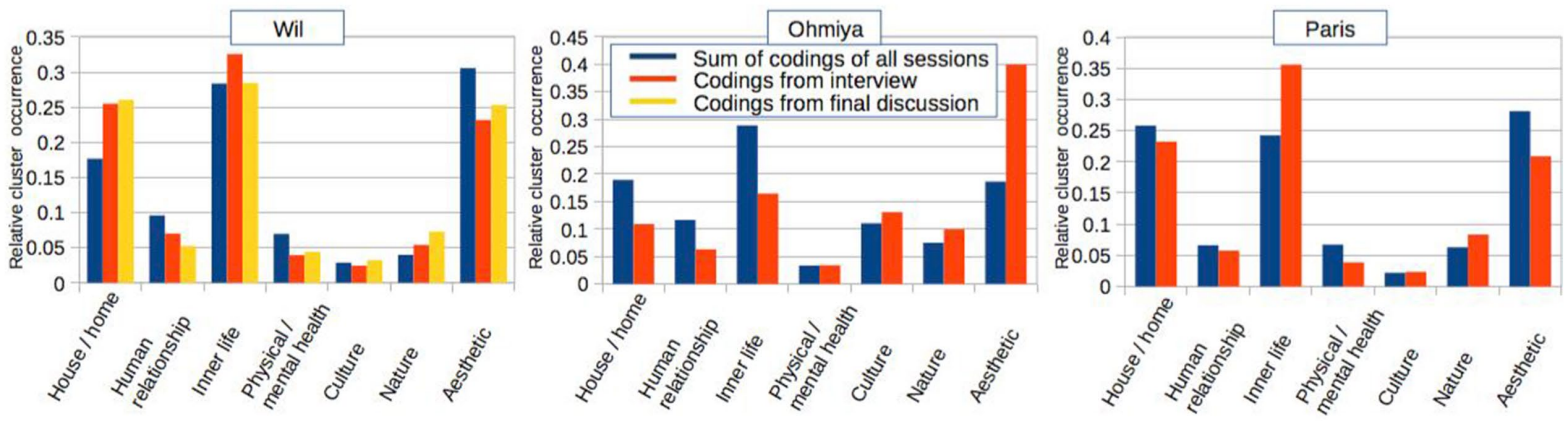
Data from the phenomenological coding and its more prominent emerged clusters regarding Wil, Ohmiya and Paris. *Blue*: coding from my observational field-notes. *Red*: coding from my semi-structured interviews. *Yellow*: coding’s from final discussions with patients (only in Wil) (Wyder, 2023).

The second step involved a clustering process of detected themes leading to patterns of data sets (1) to (3) resulting in an overarching qualitative identification of clusters and quantitative occurrences of the specific clusters, which were thereafter transformed:

- through the use of metadata to group individuals into cohorts, and
- through quantitative representation of the occurrences of the specific identified clusters in data sets 1 to 3 by metadata, such as location, gender, culture, mental and physical health, and nature (see Figure 3).

The below Figure 2 schematically shows the overall detailed analysis flow in regards to the methodological focus of this paper, whereas Figure 1 (above) is the visual representation of the overall study.

#### 2.3.7 Cross-validation of cohort-level data

An important aspect to be noted is that the quantitative data obtained by phenomenological coding (see next section) allow only a rough statistical analysis at the level of consistency among the rates of occurrence of identified emerging themes, rather than a classical statistics-based analysis. Assigning a statistical error to the rate of occurrence of themes identified in the different phenomenological data sets describing an individual patient, or to the number of themes in an identified cluster is not necessarily meaningful. Because both identifying a given textual and aesthetic element as a theme, and forming clusters from these, involves some degree of subjectivity, the resulting numerical values are affected not only by statistical but also by systematic uncertainties. Nevertheless, with sufficiently large data sets per patient, and sufficiently small numbers of clusters, it can be expected that the individual cluster occurrence rates will not vary greatly; as will be shown below, the variations between these rates for different data sets are indeed modest, thus providing a measure for the uncertainties themselves (section 4.2).

Rather unpredictably, the gathered findings’ quantitative measures serve also as an important means of validating qualitative phenomenological procedures by implementing an analytical strategy that is perhaps uncommon but, in my view, rather fruitful considering the obtained overlap of the results in respect to the different datasets. Crucially, the quantitative treatments are not there to simply ‘reduce’ the findings’ complexity and richness, nor the participants into a numerical form or conclusive ‘truth’. Rather, they must be seen as indications on a spectrum regarding specific lived phenomena that occurred during the research process, and as additional results besides the descriptive and visual findings. In that sense, this multi-facetted research strategy allowed obtaining a set of data and material that lend itself to examining - at a *quantitative* level - if (or not) correlations among the independent variables existed and permitted important quantitative cross-checking of any observed patterns and clusters.

## 3 Results

The results presented in this section stem from the overall phenomenological qualitative and quantitative procedures applied to the entirety of patients aesthetic works and narratives, together with the findings of the researcher’s observational field notes, and are now presented in quantitative terms (see Figure 3).

### 3.1 Phenomenological analysis (coding)

In regards to the present research procedure, the phenomenological analysis via coding comprised scrutinizing patients’ semi-structured recorded, transcribed and translated interviews (Wyder, 2016) as well as my observational field-notes and looking for emerging themes. These were coded with initials as, for example,

- ‘E’ for Experience
- ‘EM’ for Emotion
- ‘R’ for reality
- ‘Dr’ for Dream (night and/or daydreams)
- ‘T’ for Time

and many others. These thematic occurrences (*tags*) were thereafter regrouped into patterns leading to overall clusters by following thematic phenomenological procedures.

Based on these phenomenological groupings, a total of seven overarching *clusters* were thereafter identified, consisting of ‘House/Home,’ ‘Human Relationship,’ ‘Inner Life,’ ‘Physical/Mental Health,’ ‘Culture,’ ‘Nature,’ and ‘Aesthetic,’ Thus, each participant was attributed a numeric cluster pattern (with relative weights obtained by normalization to the total sum of the tags) based on the overall analysis of the emerged themes.

These high-level patient-specific quantitative ‘fingerprints’ correspond to the importance each patient accorded to the discovered cluster themes. These in turn are represented as tables or graphically as histograms; they represent the individual patient-based characteristics over the course of their attendance to the art therapy workshops within the three clinics. Additionally, by combining the graphs of groups of patients, this procedure also allowed obtaining an all-embracing view regarding specific patient groups. In other words, these individual or cohort-level graphs visualize the emphasis given by each patient, and each patient group, regarding the aforementioned clusters.

Hence, this procedure allowed extracting overall specificities leading to identifying commonalities among the members of a specific group or identifying differences. Thanks to the individual cluster pattern of each patient, groupings corresponding to, e.g., geographic origin and culture, but also to age, gender, PTSD-score, or other axes could be constructed and differences and commonalities along these axes corresponding to such groupings could be investigated. It should be noted that to some extent, this approach of ‘pooling’, or grouping patients by sub-groups reduces the impact of incomplete data (e.g., the shortened interview time of 30 min in the case of the Ohmiya patients), allowing subtle differences and commonalities to be detected.

To summarize, the analysis resulted in multiple levels of structured data:

- Each clinical art therapy fieldwork’s individual patient’s (e.g., cultural, self and house related) overall specificities,
- from this, personal, as well as overall patterns based on emergent themes could be identified.
- The pooling of these into three clinical fieldworks’ tables’ (Ohmiya, Paris, and Wil) forms the basis for establishing overall distinct individual, regional and cultural and house-specific self-representative patterns, together with,
- the phenomenological description, and a selection of the photographed house-based material in the form of patients’ drawings and paintings.

### 3.2 Global results

The overall analysis consequently permitted to obtain results based on individual patients, but also allows to extract clusters and cues regarding specific focus groups, and ultimately, serves to illustrate and discuss cross-cultural findings based on the European, and Japanese participants’ material and data-based findings that, added-up, result in the specific cluster patterns.

Figure 3 (see below) statistically illustrates this research’s pooled (at clinic / cohort level) findings in detail by drawing on a comparative analysis of the three principal data sets, that is the totality of coding of all art therapy workshop sessions (phenomenological observational data from the author, art therapy researcher), the coding of the semi-structured interviews (Wyder, 2016), corresponding to a quantification of an established approach, and the coding of the final interview (i.e., Wil) in regards to the three clinics in Paris, Ohmiya, and Wil (that is, the phenomenological analysis of non-structured data). Specifically, the three subfigures present for each clinical venue the total number of tags (summed over all patients of that clinic) pertaining to each of the seven emerging clusters (the ‘*cluster sum*’). The two (three) columns correspond to the three data sets of each clinical venue, and the *relative cluster occurrence* is obtained by normalizing each *cluster sum* to the total sum of cluster sums (equal to the total number of tags, Table 1) for each specific clinic’s data set.

Of particular relevance for this paper is the fact that the pooling of data for all patients of each clinic allows comparing the three data sets *within* one venue as well as *between* venues. Equally importantly, the three data sets represent phenomenological qualitative-based findings at different levels, for reasons that will be explicated in Table 1, section 4.2.

In particular, the data obtained in Figure 3 shows striking similarities within and between cohort-level occurrences of the three datasets of the three venues but also differences, which will be discussed in the following.

As discussed in section 2, attaching statistically meaningful errors to the rates of occurrence of the identified clusters in Figure 3 is not meaningful in a usual statistical sense; nevertheless, the cohort with the highest numbers of tags (Wil) exhibits the greatest degree of agreement between the three Wil data sets, while the cohort with the lowest number of tags (Ohmiya) suffers from significantly greater fluctuations between the (two) available data sets. The Paris data set lies between these two in terms of absolute numbers of tags (Table 1). The main cause for the larger discrepancies lies in the amount of data that could be gathered at each venue, which is related to the different field works durations (see 4.2).

### 3.3 Relation to study’s objectives and research questions

On a methodological level, the initial aim had been to merely carry out a phenomenological qualitative procedure that integrated quantitative aspects via PTSD-based psychometric test results, based on a rigorous and well defined protocol. That approach however proved not to be substantial enough to address the quantity and complexity of material that had emerged during the research process that spanned over several years (2016–2019). To fully analyze this material required developing a mixed-methods methodology that consisted of phenomenological qualitative and quantitative elements.

This mixed methods approach was applied in three very different venues with different socio- cultural contexts in two different types of clinics (closed, and outpatients’ wards) and with both juveniles and adults with a range of psychiatric diagnoses. In all three venues, the proposed topic of the *house* resulted in the identification of identical clusters (Figure 3), an indication firstly that the applied rigorous approach combining phenomenological qualitative and quantitative methods has broad validity but also that the proposed topic is suitable in a wide range of clinical art therapy contexts and cultures.

More importantly, the quantitative consistency between the three data sets of each venue, and crucially, between the phenomenological qualitative and the quantitative semi-structured and the non-structured interviews at each venue, is a cross-validation of the overall methodology. Given the difficulty of validating phenomenological approaches, the observed consistency is an unexpected but very important substantiation of the phenomenological elements of the complementary approaches. This substantiation could only be established thanks to the size of the data sets and the quality and richness of the obtained data. It also underlines the suitability and reliability of the applied method and the importance of the rigor of the observations and subsequent note taking.

### 3.4 Complementarity between quantitative and qualitative findings

The quantification (through coding) of the qualitative (phenomenological observational), the classical (semi-structured interview), and unstructured (free-form interview) data has allowed to compare the information content of the three data sets which appear to result in very comparable outcomes, in that high level patterns emerge at similar rates (Figure 3). This quantification provides a metric, but ‘only’ reflects patterns that are already present in the qualitative data. Yet without this quantification, these patterns might have been suspected, but could not have been extracted and made apparent with the same level of confidence. It is the *combination* of these two approaches that results in a coherent view and that furthermore allows identifying structures (e.g., the relatively greater importance of the cultural cluster for the Japanese patients) that point towards the need for deeper investigations (paper in preparation).

Also, and importantly for the field of art therapy, the carried out procedures allowed demonstrating that findings and data stemming from such research do indeed allow objective outcomes that are beyond art therapy researchers’ own ‘subjective’ and ‘intuitive’ interpretation.

#### 3.4.1 Merging of qualitative phenomenological and quantitative approaches

The complementary roles that qualitative and quantitative data play must be seen, I argue, within a larger context. Unfortunately, and according to Knappertsbusch (2023, p. 19) a ‘qualitative-quantitative-divide largely persists despite the growing relevance of integrative approaches in social research methodology’. In contrast, the present adopted qualitative phenomenological and quantitative research methodology needs to be regarded as non-hierarchical, that is, the overarching fine-grained level of phenomenological qualitative methodology lies at the heart of the detailed and complex quality of the observations and the resulting descriptive, pictorial, as well as quantitative findings.

Hence, the purely quantitative procedures, as stated earlier, have to be seen as additional and complementary ways of gathering data (e.g., PTSD test results), while phenomenological coding (van Manen, 2011) and their analysis contain elements of both qualitative phenomenological and quantitative approaches. In combination, the herein applied methodology allowed to provide art therapy-related findings such as, for example, patients’ long-term art therapy workshop fieldwork evolutions, or the possibility of fieldworks-based overall data pooling, which permitted detecting patterns and themes that would have been difficult to discover and analyze otherwise. Crucially, and as will be shown in section 4, this mixed-method approach of including phenomenological qualitative and quantitative procedures led to a quantitative validation of a phenomenological qualitative research strategy.

To summarize, my approach consisted of a combination of both qualitative phenomenological and quantitative methodologies in which each carries a similar weight, even though phenomenology is the overarching philosophical stance in which the quantitative procedures are embedded. This combined approach has not only yielded rich data, but has also allowed extracting information at the individual, cohort and cross-cohort level, which on one hand has validated the phenomenological data-collection procedures, and on the other hand has allowed cohort-level and inter-cohort level specificities to be identified for further studies (e.g., culture in Japan).

## 4 Discussion

The development of a dedicated mixed methods methodology allowed the multiple aspects covered in this research to be addressed and permitted cross-checking a phenomenological qualitative enquiry applied to an art-therapy approach. As will be discussed below, this can be considered a validation of the qualitative phenomenological procedures through quantitative measures.

Importantly, given the sizes of the available datasets, commonalities and differences on a cohort-level could be evaluated and validated quantitatively, which allowed determining whether differences among rates of occurrence can be considered relevant or not; the agreement between the three (two) cluster rates of each venue (better for larger data sets, Table 1) is a quantitative measure of this agreement.

### 4.1 Cross-validation of mixed-methods procedures: appearance of identical clusters

Figure 3 (Wyder, 2023) summarizes in graphical form the quantitative outcomes of the phenomenological coding procedures (phenomenological observations versus semi-structured interviews (Wyder, 2016)), applied to aesthetic works and narratives of the three fieldwork venues in Paris, Will and Omiya. As shown by the agreement between the rate of occurrence of each cluster in the three (two) data sets within each venue (Figure 3), the following clusters were independently found to arise from the phenomenological coding procedures applied to the three data sets, and this for each venue:

- House / Home
- Human relationship
- Inner life
- Physical/Mental Health • Culture
- Nature
- Aesthetic

These clusters and their relative occurrences identified the principal concepts and their importance for the three patient groups. The implications of the methodological findings are discussed in more detail in the following while highlighting some examples. Crucially, the consistency between the findings obtained via qualitative phenomenological approaches and the data based on the evaluation and analysis via quantitative procedures demonstrates and substantiates the validity of phenomenological observational field-note taking (narratives and aesthetic works) and of the following coding procedure (van Manen, 2011); these allowed discovering emerging themes and patterns leading to identical clusters as interview-based approaches and are thus an integral and accurate part of a qualitative phenomenological scientific method.

**Table 1:** Data summary for the three clinical groups: cohort sizes, duration of the interventions, and overview of the tag data sets obtained through coding of the different observational data sets (see text).

Clinical venue	Number of patients	Number of tags (observational fieldwork session notes)	Number of tags (semi-structured interviews)	Number of tags (final discussion)	Duration (months)
Wil	7	2′513	1′929	1′086	5
Paris	6 (4) *	953	833	-	3
Ohmiya	9 + 6	987	413 **	-	1

*Two of the six patients were excluded due to a very brief workshop attendance. **The semi-structured interview (Wyder, 2016) had to be shortened to a maximum of 30 min consisting of 12 of the 29 questions. Also, a relatively high number of patients did not take part in the interview due to drop-out.

### 4.2 Quantitative validation of the overall qualitative findings

Remarkably, from a methodological and phenomenological point of view, the coding-based comparison of the data in Figure 3 demonstrates a strong coherence between the two (or three, in the case of Wil) types of datasets for all fieldwork venues. This means that the carried out analytical method permitted a crosscheck of each type of qualitative phenomenological and quantitative method and therefore additionally permits a cross-validation of each one. Thus, the similar values of the relative cluster occurrences within clusters, and this for all fieldwork locations, underscores the quantitative consistency of the different qualitative phenomenological approaches.

This consistency of the obtained findings on cluster occurrence rates within each of the three venues for three (two) different types of data sets demonstrated in Figure 3 (Wyder, 2023) ultimately shows that the outcome of all the carried-out procedures is on one hand independent of the specific approach, and on the other, indicates that the observed clusters and their relative weights have a degree of objective validity. For instance, the overall semi-structured interview (Wyder, 2016) based findings regarding the themes and clusters demonstrate a strong similarity to the emerged themes and clusters stemming from my observational field-notes. This information is important because both of these data sets are part of an ensemble of findings that stem from a combination of patients’ narratives, patient-based individual semi-structured coded verbal expressions, and my observational field-notes (narratives and aesthetic works’ descriptions).

Note nevertheless the imperfect concordance of the two different approaches within, for example, the ‘Aesthetic’ cluster in Ohmiya, or ‘Inner Life’ cluster in Paris which highlights the limitation of a purely quantitative interpretation of the data, and thus relativizes a ‘belief’ in greater trustworthiness of quantitative results. At the same time the imperfect agreement between the relative rates as determined by the three different approaches, that is, the phenomenological observation, semi-structured interviews (Wyder, 2016), and final discussions in Wil, provides a measure of the uncertainty of the individual findings of the coding. Thus, phenomenological coding of the differently obtained datasets, while agreeing on the gross features of the relative importance regarding the identified clusters, needs nonetheless to be regarded as indicative, pointing to specific trends and clusters within a spectrum.

With this small caveat, the applied analytical strategy can nevertheless be considered as a methodological step forward in art therapy research, as it allows to cross-validate a phenomenological qualitative and an interview-based qualitative approach resulting in the identification of identical conceptual clusters and furthermore in the same proportions. Furthermore, the concordance between the three approaches is best for the venue with the richest data set due to this fieldwork’s longest duration (see Wil, Table 1). Importantly, the overall finding of consistency is also independent of the involved cultures, ethnicity, age, gender, or the psychiatric clinics’ closed, and out-patients’ format. It is vital to keep this result in mind given the ethnic and cultural diversity in contemporary societies’ that are reflected within a psychiatric context.

The detailed data that form the basis of Figure 3 are presented in Table 1, which summarizes on one hand, the size of the three clinical groups and the duration of the interventions, and on the other hand, the size of the *tag* data sets obtained through coding. These are provided in form of the total number of identified individual thematic occurrences, or *tags, summed over all patients of a specific venue,* for the two (three) different data modalities obtained at each venue. The thematic occurrences (tags) obtained for each individual are grouped and combined per venue, at which point overarching venue-level clusters are identified. The relative rates of occurrence of these overarching clusters are shown in Figure 3 for each venue and for each data modality (in Figure 3, the groupings are by venue, but other groupings are possible).

The detailed numbers of Table 1 allow to shed light on the contrasting differences between the different datasets, especially those concerning Paris and Ohmiya in terms of the different fieldwork durations (Paris 3 months, Ohmiya 4 weeks). Based on the results delineated in Figure 3 and Table 1 it can be stated that the 5 months long clinical art therapy fieldwork in Wil allowed gaining the most detailed results, in contrast of the fieldwork venues in Paris and Ohmiya. Additionally, the Wil results demonstrate the rather convincing agreement between the different datasets based on the aforementioned methodology, based on the researcher’s own observations including patients’ narrative and aesthetic works, those stemming from patients’ interviews, and in regards to the findings of the final discussion to Wil. Variations in Figure 3 among modalities for a given cluster and venue are a reflection of the amount of data obtained for each patient (Table 1); shorter clinical durations and reduced interview durations result in less tags being identifiable, and consequently greater fluctuations of the cluster rates based on these.

A further aspect must also be taken into account: cultural differences, in particular regarding self-expression, can also easily lead to lower rates of occurrence of clusters related to individuals’ inner life and personal identity, and higher rates of more ‘neutral’ themes, in a ‘formal’ interview context than in an informal group setting. Also, in the case of Ohmiya, there was presence of a mental health worker in both groups, which might have influenced patients’ utterances in being more, or less explicit.

Finally, while the relative rates of the identified clusters are globally comparable among the three venues, the sensitivity of the quantitative procedure is such that it allows identifying even small differences between cohorts, such as the enhanced importance of the culture cluster in the Ohmiya cohort. This topic is discussed in depth in a paper under preparation.

### 4.3 Research methodological findings

This study’s findings demonstrate that if a rigorous qualitative phenomenological method is applied within art therapy research, the resulting quantitative findings can be considered as valid, as shown by the agreement of cluster occurrence rates within cohorts in Figure 3. Equally importantly, the identification of identical clusters for different cohorts underlines the universality of the chosen approach. This validation of the phenomenological approach through the agreement of its observations with the interview-based approaches has several consequences on art therapy research methodologies, and on the reliability of the art therapeutic practice if rigorously applied.

Furthermore, an exclusive textual description of findings is often difficult to grasp., and one might get lost in the mounds of textual data. Consequently, going beyond ‘usual’ art therapy and qualitative phenomenological procedures by adhering not only to a quantitative analysis in terms of theme and pattern detection, but also applying graphical presentations of the findings, allowed to seize these more easily as it provides an intelligible overview. In that sense, the resulting numbers are by no means regarded as a reduction, or demotion of the persons involved, nor do they claim to indicate an absolute, or increased level of ‘precision’ and ‘exhaustiveness’. Instead, my procedure must rather be regarded as an expansion of, or association between, the previously validated procedures in the fields of art therapy research and phenomenology by including quantitative approaches.

Importantly, this study represents a significant advance over the existing published literature on art therapy research methodologies, as both the form of this study as well as its conclusions cover new ground. An art therapy literature review (albeit only in English, French and German) demonstrated that similar research (with even partial overlap) to the present one spanning over several psychiatric, and socio-cultural contexts, addressing aesthetic and verbal symbolic representation (in the present study, based on the theme of the house) investigating notions of patients’ selves, and employing multiple data collection modalities simultaneously seem to be absent. This is not to say that earlier studies have not covered similar ground: quantitative attempts at evaluations of phenomenological investigations (but not in art therapy contexts) are seeing recent interest (e.g., Szula et al., 2024; Liu, 2024; Fernandez Velasco et al., 2023), and in the field of art therapy (Van Lith et al., 2023), a range of mixed-methods research methodologies have been investigated in the last decades, among them several based in phenomenology (Gerber et al., 2024), which however did not have an emphasis on quantitative results.

However, no prior art therapy research appears to have carried out a quantitative evaluation of the obtained phenomenological qualitative findings; it is the combination of those two elements that was critical to reaching the conclusions of this study. As highlighted in the introduction, the integration of phenomenology is not new in the field of art therapy, nor is the integration of quantitative measures via psychometric tests. However, the usage of quantitative measures as applied to phenomenology in this art therapy study can be considered a paradigm shift in art therapy research as it allows cross-checking, and as it were, validating, albeit not in the usual statistical sense, the multitude of findings and patient observations. Importantly, this is a very different aspect than quantitative strategies carried out in order to investigate the effectiveness of art therapy (e.g., Saunders and Saunders, 2000).

As pointed out in Table 1, and discussed in 4.2, more accurate data and better agreement among different types of patient data is provided by longer duration phenomenological observation. Given this agreement between phenomenological observation data and data obtained by more classical quantitative methods, one might consider partially replacing the later with the former.

### 4.4 Practical implications

#### 4.4.1 Art therapy practice-based recommendations

Although the focus of this paper lies on methodological questions, some implications for art therapy practice can be highlighted, in particular as this study has shown the suitability of the topic of the *house* in different cultural contexts, but also because of the importance of cultural awareness for phenomenological observation:

- Theory must be appropriately adjusted to idiosyncratic cultural context: meanings of symbolic expressions cannot be assumed to be universal, but equally, extrapolation from a given culture to others requires cultural sensitivity, awareness and specific knowledge.
- The choice of a topic (if applicable) that is an inextricable part of all cultures and that allows integrating the self can be considered a specific art therapy ‘tool’. Strongly emotionally loaded and encompassing topics allow the investigation of the central assumption of art therapy that symbolically charged aesthetic work and individualized narratives produced in the context of art therapy can help to externalize, address, and transform personal issues *because* patients’ expressions are personally shaped, leading to (ideally) individualized change, and inner transformation.

#### 4.4.2 Methodological recommendations for art therapy research

Adopting the methodology of this study enabled gathering complex and multi- facetted material and offered the possibility of not only an in-depth analysis, but of additionally ‘doubly’ scrutinizing the obtained data, which increased the overall validity of the results. Findings based on such a mixed-method approach might also be more accessible and appropriate to other mental health professionals such as psychiatrists, or psychologists. Two aspects can consequently be considered as important for art therapy research:

- To consider a mixed methods approach, which permits gathering rich qualitative and quantitative data that serve to obtain material from diverse sources,
- and to carry out several analytical strategies, which allow crosschecking the obtained data, which increases the findings’ validity.

A further methodological recommendation is to theoretically anchor a predefined art therapy, or research approach that is both matched to one’s personal stance, but also appropriate to investigate the formulated research question. This requires being aware of, and open to, what the specific art therapy research situation demands, which includes integration of oneself, the patients, the socio-cultural, clinical, and geographical contexts. Hence, in my view, scientific research in art therapy requires constant verification, adaptation, and potential change of initial preconceived pathways in order to do justice to a particular research question, situation, and to all persons involved, which asks for integrating a self-reflexive stance.

Finally, following rigorous, systematic, and as equally replicable as possible research procedures is fundamental for gathering the most consistent material even if this may prove to be challenging under diverse fieldwork situations and cultural specificities. Yet, such a methodological approach lends itself to realize comparative (e.g., cross-cultural) studies that allow identifying specific trends and patterns within each fieldwork situation, and allow to thereafter draw reliable conclusions at the individual level or for well-defined sub-groups.

## 5 Conclusion

### 5.1 Most salient findings

The cross validation of the applied mixed-methods procedure demonstrated the equivalence and objectivity of the different qualitative phenomenological approaches in quantitative terms through the identification of identical conceptual clusters and in the same proportions within each of the three clinical venues. Crucially, the consistency between the findings obtained via qualitative approaches and the data based on the evaluation and analysis via quantitative procedures demonstrates and substantiates the validity of a phenomenological qualitative approach, and of the following coding-procedure (van Manen, 2011); altogether, these allowed discovering emerging themes and patterns leading to clusters that are an integral part of a qualitative phenomenological scientific method.

This study’s findings demonstrate that the topic of the house was used by all of the patients as a pictorial, narrative and symbolic form of self-representation irrespective of the socio-cultural context, or the clinical format in Japan, France and Switzerland, and can thus be considered as a valid art therapy proposition. This theme was also a valid proposition cross-culturally irrespective of patients’ socio-cultural, ethnic, psychopathological, age- and gender-related backgrounds. This thematic suggestion, the *house*, incorporates a very potent emotive stimulus, which allowed a wide range and depth of symbolic individualized patient-based topical expressions that permitted patients and art therapist and researcher to carry out individualized sensitive therapeutic work, including the present research results.

### 5.2 Implications for research and practice

As research in art therapy is still rather uncommon, the first implications for art therapy practice are that art therapy formation should include studies in phenomenology and according suitable training in observation, note- taking and analysis, but also an awareness of the complementary roles that quantitative and qualitative approaches can play. In that sense, instruction regarding an understanding of quantitative methods would also be recommended. It would also be desirable if art therapy research that includes quantitative elements could be disseminated more broadly within the community.

In practical and clinical terms, an understanding by clinicians that phenomenological observations stemming from art therapy have clinical validity and can complement other forms of data collection is desirable; a more complete picture of the patients’ inner state that would otherwise require administration of more formal interview-based, or ‘interrogatory’ approaches would result.

Naturally, confirming the results of this study with other groups should have priority, ideally building on long therapeutic interventions, which yielded the most reliable data in this study. For consistency purposes, such groups should ideally have characteristics comparable to those of this study, but investigating additional groups with other characteristics should also be pursued, as it would provide complementary reinforcing (or contradicting) evidence for the observations of this study. Such research requires of course appropriate training of the researchers regarding a mixed methods approach, but also in observational and documentational techniques; this would then open the door to exploring the impact of the observing on the outcome of a mixed-methods study.

### 5.3 Study’s strengths and limitations

It is evident that the complexity of this cross-cultural study also entails limitations. For example, the three functions I constantly occupied–that of an art therapy researcher, of the patients’ art therapist, and that of self-observer–can introduce a limitation to this research, since exercising several roles simultaneously represented a challenge to equally accommodate each one. These multiple roles could have resulted in a certain level of incompleteness and subjectivity, which was however unobtainable without the co-presence of the other roles.

Also, limitations subsist due to publications-based literature searches, which mostly scrutinized English, but also French and German literature. ‘Unorthodox’ methods of searching and discovering topic-related literature outside formal academic channels turned out to be crucial and allowed gathering valuable additional, often unexpected material within various cultural contexts.

Moreover, limitations subsisted regarding the number of participants in these field-works and the rather dissimilar fieldwork periods and diversity of patients’ attendance to the art therapy workshops. Findings have thus to be regarded as indicative. Nevertheless, even though the research periods, patient ages, clinical approaches and cultural contexts were varied, the strength of this study is the consistency among the outcomes of the carried out mixed methods, that is the phenomenological qualitative and quantitative methodology. In that sense, as Figure 3 shows, the agreement of the different data sets’ in the cluster-based results demonstrate that any potential biases seemed to be minor.

Nevertheless, in spite of these limitations, this study demonstrates that if a rigorous phenomenological qualitative *and* quantitative method is applied within art therapy research, the resulting overall qualitative findings can be considered as valid, as the carried-out quantitative cross-checks demonstrated.

## Data Availability

The original contributions presented in the study are included in the article/supplementary material, further inquiries can be directed to the corresponding author.
